# Increased long-term central memory T cells in patients with retreatment pulmonary tuberculosis

**DOI:** 10.3389/fimmu.2025.1545537

**Published:** 2025-03-18

**Authors:** Xin Yao, Haomin Cai, Jianxia Chen, Fangyong Yu, Xiaocui Wu, Yarong Shi, Yang Hu, Yuyan Xu, Qinghua Xu, Zhonghua Liu

**Affiliations:** ^1^ Shanghai Key Laboratory of Tuberculosis, Shanghai Pulmonary Hospital, Tongji University School of Medicine, Shanghai, China; ^2^ The Key Laboratory of Environmental Pollution Monitoring and Disease Control, Ministry of Education, School of Public Health, Guizhou Medical University, Guiyang, China

**Keywords:** T lymphocyte subsets, center memory T cells, retreatment tuberculosis, efficacy, immunotherapies

## Abstract

**Background:**

T cells are crucial in controlling Mycobacterium tuberculosis infection and disease progression. Nevertheless, the specific functions and changes of T lymphocyte subsets in retreatment tuberculosis remain poorly understand. The study aims to identify the changes in T lymphocyte subsets and the immunoprotective effect of T_CM_ in retreatment tuberculosis.

**Method:**

We collected venous blood from the participants and assessed using flow cytometry. Univariate analysis and regression model were used to evaluate the changes of T lymphocyte subsets and key subsets in retreatment tuberculosis.

**Results:**

In the study, while the frequencies of CD4 and CD8 T cells were similar between primary and retreatment patients, retreatment patients exhibited a significant increase in T_CM_ (*P* < 0.05), which may represent a protective factor for retreatment (adjusted OR=0.926, 95%CI: 0.860-0.996, *P* < 0.05) (adjusted OR=0.951, 95%CI: 0.912-0.992, *P*<0.05). Furthermore, T_CM_ significantly increased in retreatment patients who achieved cure (*P* < 0.05), though were similar between the cure and no-cure for primary patients; The potentially protective effect of T_CM_ in patients with repeated infection may possibly contribute by improving the efficacy of retreatment chemotherapy (adjusted OR=0.803, 95%CI: 0.677-0.953, *P* < 0.05) (adjusted OR=0.890, 95% CI: 0.812-0.976, *P*<0.05), particularly in those with lung injury (adjusted OR=0.780, 95% CI: 0.635-0.957, *P*< 0.05) (adjusted OR=0.805, 95% CI: 0.660-0.983, *P*<0.05).

**Conclusion:**

Development of adjunct immunotherapies for increasing T_CM_ responses may improve the efficacy of retreatment tuberculosis with existing and with novel chemotherapies.

## 1 Introduction

Tuberculosis (TB) remains the leading cause of death caused of a single infectious agent (1). The emergence of drug resistance (DR-TB) has increased the difficulty of TB prevention and treatment, making patients higher retreatment risk. T cells play a critical role in containing of Mycobacterium tuberculosis (Mtb) infection and reinfection, and immunotherapy has shown promise in enhancing T-cell responses (2). To effectively reduce the disease burden associated with DR-TB and retreatment tuberculosis (Re-TB), it is essential to deepen our understanding of T-cell dynamics and the contributions of key subsets in the context of Re-TB. Re-TB is defined as the recurrence of symptoms or signs of active TB in patients after treatment failure or more than one month after the end of treatment (3).

For decades, T-cell immune mechanisms involved in clearing Mtb have been studied in both human and animal models, highlighting the significance of T cells in TB drug development and immunotherapy (4, 5). The immune function of T cells in TB is primarily reflected in cytokines like IFN-γ, secreted by activated CD4 and CD8 T cells, which mice and humans with IFN-γ deficiencies or abnormalities in IFN-γ receptor show increased susceptibility to TB (6, 7). Additionally, Mouse models have demonstrated that the absence of CD4 T cells leads to greater susceptibility to TB compared with wild-type mice; however, lung infections can be controlled and stabilized in the absence of CD8 T cell-mediated immunity (8). Patients with active TB often experience immune dysregulation due to prolonged inflammation, resulting in a reduction in CD4 and an increase in CD8 T cells (6, 8, 9). Previous clinical studies in adults with normal immune function have shown that the sputum smear grade at diagnosis can predict the relapse and retreatment of TB (6). However, it remains unclear whether T-cell immunity function is stronger in Re-TB with long-term infection. A mouse model has demonstrated that in relapsed TB memory Th-1 expanded and aggregated in the lung, efficiently controlling Mtb growth (10). Additionally, the upregulation of BTLA on CD4 and CD8 has been observed in Re-TB, leading to T-cell exhaustion in chronic infections (11).

Immunological memory, primarily mediated by memory lymphocytes, is the foundation of vaccine administration and the key to preventing relapse and retreatment in TB patients (12). Memory lymphocytes are a heterogeneous population that can quickly generate strong immune responses by dividing into two functional subsets, defined by CCR7 and CD45RA: central memory T (T_CM_) cells and effector memory T (T_EM_) cells (13, 14). T_CM_ cells, first identified through human immunology studies in 1999, have been shown to exhibit heightened immunological activity and memory to play a crucial role in anti-infection responses (15, 16). T_CM_ cells are long-lived cells with self-renewal and proliferation capabilities (17). When the host is exposed to foreign antigens, they quickly activate, migrate to the lymph nodes, and initiate the antibody response, thereby providing protective immunity against infection (18, 19). Studies in guinea pig models and pharmacological research have shown that an increase in T_CM_ contributes to the effectiveness of the BCG vaccine (20, 21). Nevertheless, the mechanisms by which immunological memory, particularly T_CM_, exerts a sustained enhancing effect on immunity against the same pathogen during relapse and retreatment remain poorly understood.

At present, a large body of research has confirmed the immune role of T cells in active pulmonary tuberculosis (PTB). However, there are relatively few studies on the changes in T lymphocyte subsets in Re-TB, and the protective effects of immunologic memory during retreatment remain unclear. The aim of the study is to analyze T lymphocyte subsets in Re-TB, which may provide valuable insights for enhancing immunotherapy and developing therapeutic vaccines for Re-TB in clinical practice.

## 2 Method

### 2.1 Ethics statement

Our experiments were in accordance with the ethical standards formulated in the Helsinki Declaration. This study was approved by the Ethics Committee of Shanghai Pulmonary Hospital, Tongji University School of Medicine (Shanghai, China) (K19-060Y). All participants gave verbal consent for the use of their clinical information for research purposes.

### 2.2 Study paticipants

**Figure 1:**
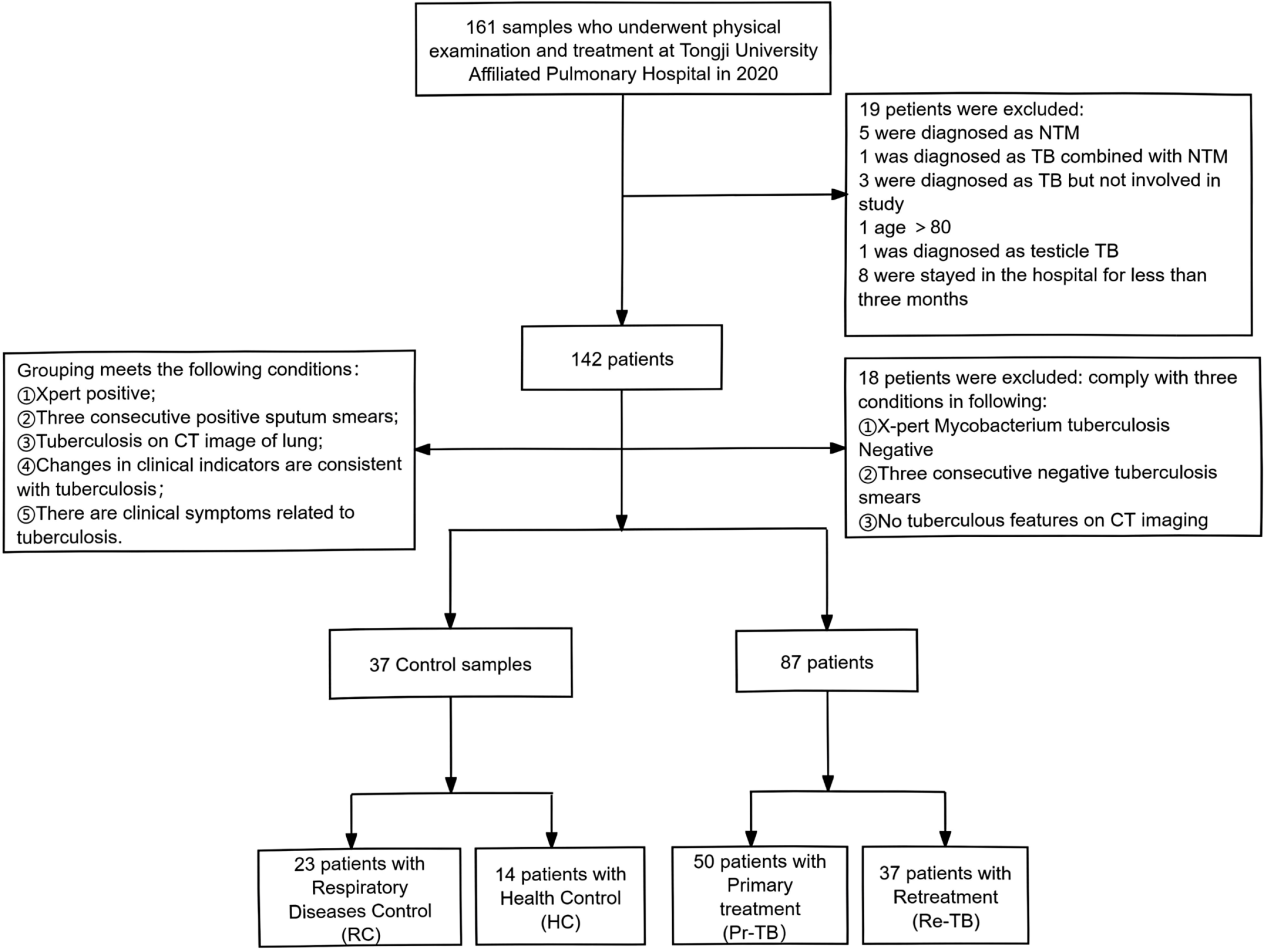
Flowchart of paticipants included in the study. HC, Healthy Control who were not found to have any illnesses during the physical examination; RC, Respiratory Control without TB but diagnosed with other respiratory system diseases (Pneumonia, COPD, cancer, etc); Pr-TB, Previously not infected with Mtb; Re-TB, Including patients with recurrance or retreatment, recurrance refers to patients who terminated treatment and relapsed again after six months of discontinuation.

In the study, we recruited 124 participants from Shanghai Pulmonary Hospital in 2020 (**Figure 1**), all participants have not received vaccination recently. We collected whole blood samples from the participants and extracted their baseline and clinical information from the hospital’s electronic case system, monitoring their treatment over two years. The data were then used to establish a database, which included the participants’ gender, age, height, weight, admission information, clinical diagnostic results (sputum smear, CT, etc.), and treatment outcomes. **Supplementary Table 1** provides a summary of the demographic data of the subjects included in the study and the X-pert results. The study is based on testing a relatively small group of 37 patients with retreatment pulmonary tuberculosis.

### 2.3 Sample collection

Venous blood was collected from participants before treating, with 20ml of blood draw from both the PTB and non-TB using heparin anticoagulant tubes (BD Biosciences, US). The samples were then labelled and processed according to the manufacturer’s instructions (Biosciences). Immunophenotyping of CD4 and CD8 T lymphocyte subsets was performed within a few hours of sample collection.

### 2.4 Data collection and definition

In accordance with the definition and guidelines for TB published by the World Health Organization (WHO) (3), treatment is considered complete when at least two negative bacteriological tests are obtained, indicating a cure. In contrast, positive Mtb tests or the absence of clinical improvement are considered indicative of treatment failure (no cure). Pr-TB is defined as a condition in which the patient has not been previously infected with Mycobacterium tuberculosis (Mtb). Re-TB primarily includes patients with recurrence or retreatment; recurrence refers to patients who relapse after completing treatment, with a gap of more than six months since discontinuation; retreatment pertains to patients who have stopped taking medication for over six months following treatment failure, mainly rifampicin. The stained slides are examined under a microscope to detect acid-fast bacilli (AFB), and the number of AFB observed in a given number of fields is used to classify the smear as follows: (1) Negative: No AFB observed in 100 fields. (2) Positive: Including 1+, 2+, 3+, and 4+ categories; 1+: 1-9 AFB per 100 fields; 2+: 1-9 AFB per 10 fields; 3+: 1-9 AFB per field; 4+: 10 or more AFB per field.

Chest computed tomography (CT) data for PTB patients (n=87) were collected in the electronic system of the Shanghai Lung Hospital for the first treatment. A scoring scale (**Supplementary Table 2**) was developed based on the methodology used by Anna P. Ralph et al. (22) for scoring CT in PTB, with a total score of 15 points. A visual assessment of the CT images was performed, with lesions graded according to the following criteria: (1) <3: absence of lung injury; (2) ≥3: presence of lung injury. The scoring is illustrated in **Supplementary Figure 1**.

### 2.5 Flow cytometry

The blood was centrifuged to separate the serum, then re-suspended in pre-cooled buffer (BD Biosciences, US) and centrifuged at 4°C to obtain a cell suspension. The final concentration of the cell suspension was adjusted to 10^7^ cells/ml using the pre-cooled buffer. A 100 μl aliquot of the prepared cell suspension was transferred into a 12-round polypropylene tube, and an appropriate amount of specific surface antibodies (CD3, CD4, CD8, CD45RA, CCR7, CD27, CD28, CD57, PD1) was added to each tube. After 30 minutes of incubation on ice, the cells were washed twice with buffer and centrifuged at 4°C, then gently mixed them. Cytoflex S (Beckman-Coulter) was used to record at least 20,000 events/samples. Multi-parameter flow cytometry data were analyzed using CytExpert. In flow cytometry, cells were first gated based on forward and lateral scatter plots to identify the lymphocyte population, followed by selection of live cells, and then monomorphic cells. Adjust parameters through single-color staining and set up a cross or rectangular gate to distinguish between negative and positive cell populations. The selection of antibodies and gating for different T lymphocyte subsets is described in **Supplementary Table 3**. Data for the cell population were recorded as percentage of cells.

### 2.6 Statistic analysis

Statistical analysis was performed using SPSS 26.0 and GraphPad Prism 9.5. Differences in baseline demographics (categorical data) between groups were analyzed using the χ2 test or Fisher’s exact test. Differences in T lymphocyte subsets (count data) were analyzed by performing statistical tests with SPSS 26.0 for subsets analysis, while GraphPad Prism 9.5 was used to graphically display the results, and FlowJo 10.8.0 was employed to visualize cell subsets. The distribution differences of subsets among the four groups (HC, RC, Pr-TB, Re-TB) were analyzed using either ANOVA or the Kruskal-Wallis H test, with Bonferroni correction applied for multiple comparisons. The differences in subset distribution between the two groups (Re-TB and Pr-TB) were analyzed using either the t-test or the Wilcoxon signed-rank test. The Kaplan-Meier method was used to analyze the cumulative cure rate of tuberculosis patients. Logistic regression is used to analyze univariate and multivariate data by predicting the probability of the presence or significance of different T cell subsets, offering a seemingly simplistic approach to exploring their relevance in immune responses to such an important pathogen due to their synergistic functions. P < 0.05 was considered statistically significant.

## 3 Results

### 3.1 Initiation of T-cell immunological abnormalities in retreatment tuberculosis

**Figure 2:**
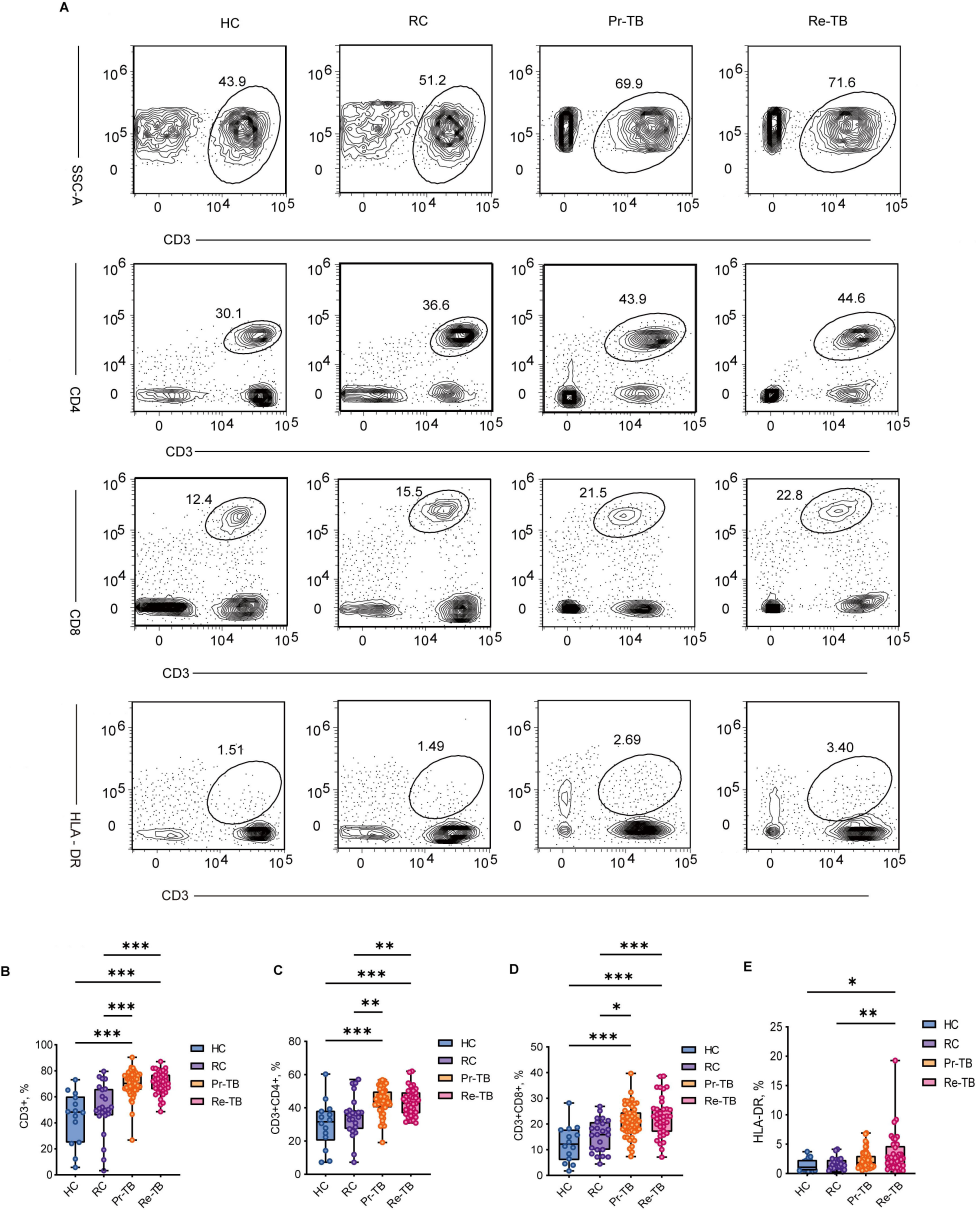
Frequency of main circulating T cells in different groups. **(A–C)** Representative dot plots of Mature T lymphocytes(CD3+) **(A)**, Helper T lymphocytes(CD3+CD4+) **(B)**, Cytotoxic T lymphocytes(CD3+CD8+) **(C)**, HLA-DR **(E)** subsets showing the mean frequencies of each group. Inter group comparison data follows a normal distribution and is analyzed using analysis of variance. Otherwise, Kruskal-Wallis H test is used, followed by Bonferroni test to correct for multiple comparisons. Significant differences are expressed as *<0.05, **<0.01, ***<0.001. HC, Health Control (n=14); RC, Respiratory Disease Control (n=23); Pr-TB, Primary treatment tuberculosis (n=50); Re-TB, Retreatment tuberculosis (n=37).

Study has shown an increase in CD4 and CD8 T cells in active TB. However, the changes in T cells in Re-TB remain poorly understood, consequently, we compared them to those in three other groups. Our results revealed that CD3 T cells were significantly higher in Re-TB patients than in individuals with HC and RC (*P*<0.001) (**Figures 2A, B**). Compared to HC and RC, there was a significant increase in CD4 and CD8 T cells in Re-TB and Pr-TB (*P*<0.05) (**Figures 2C, D**). We also observed that HLA-DR, a marker of T-cell activation, was significantly increased on CD3 T cells in Re-TB patients (*P*<0.05) (**Figure 2E**). Notably, these differences in T cells were exclusive to comparisons between TB and non-TB, with no significant differences observed between Re-TB and Pr-TB, nor between RC and HC.

### 3.2 Decreased T_Naive_ and increased T_CM_ in retreatment patients

**Figure 3:**
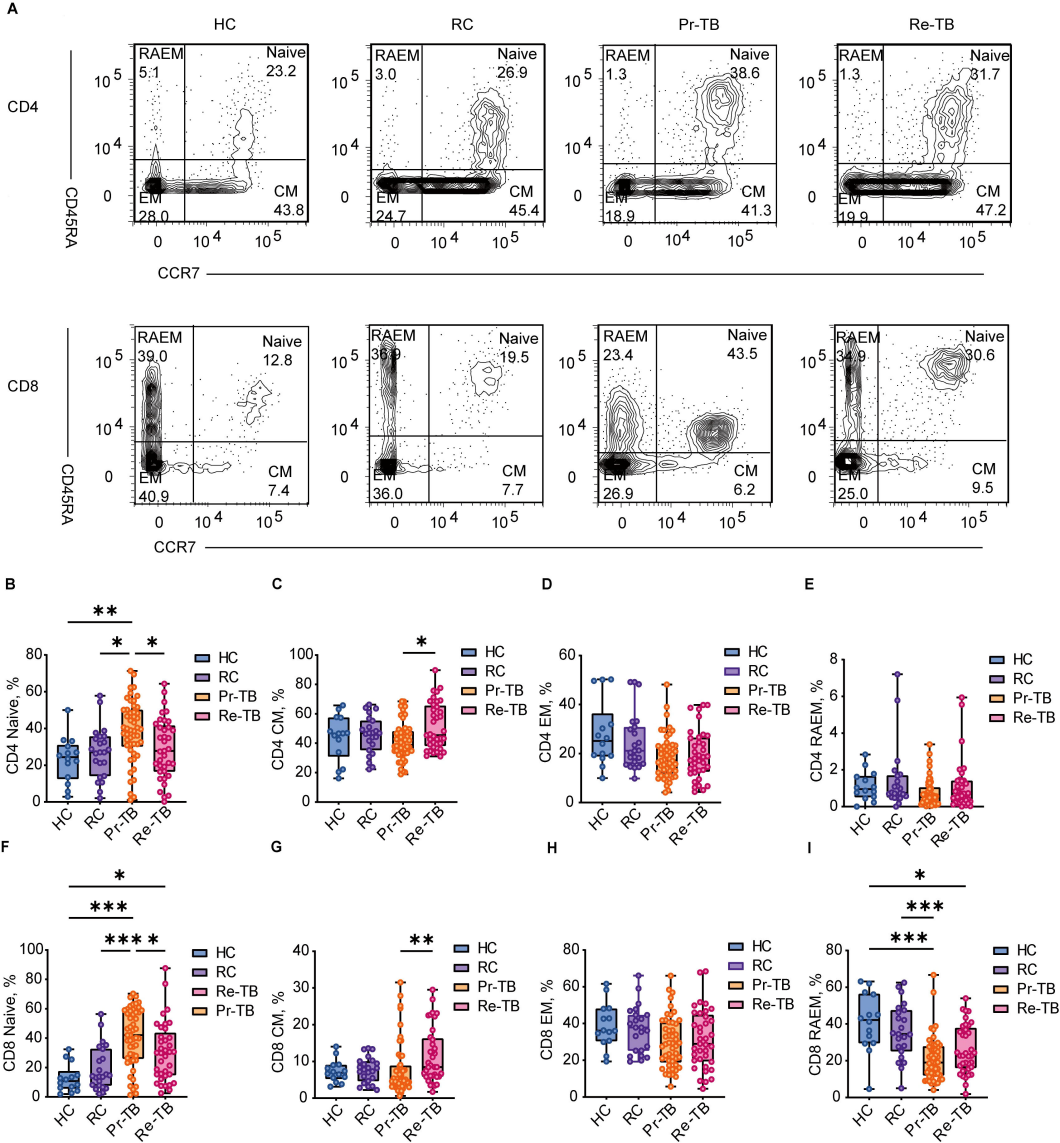
Frequency of CD4+ and CD8+ T lymphocyte subsets with different patterns of CD45RA and CD62L expression in different groups. **(A, B)** Representative dot plots of Naive(CCR7+CD45RA+), Central memory(CM, CCR7+CD45RA-), Effector memory(EM, CCR7-CD45RA-), RA-Effector memory(RAEM, CCR7-CD45RA+) cell subsets showing the mean frequencies of each group. **(C–F)** Scatter plot showing the frequency comparison of CD4+ in different groups. **(G–J)** Scatter plot showing the frequency comparison of CD8+ in different groups. Significant differences are expressed as *<0.05, **<0.01, ***<0.001.

CD4 and CD8 T cells undergo further differentiation to control the progression of Mtb infection and reinfection. Research has shown a significant increase in T_Naive_ cells during active TB. During active bacterial replication, there is a proliferation of T_EM_ cells, while T_CM_ cells are predominantly observed after the infection has been controlled or eradicated. It has been suggested that T_CM_ cells may serve as a valuable biomarker for differentiating between LTBI and active TB. To investigate changes in T lymphocyte subsets in Re-TB, we examined four subsets across four groups (**Figure 3A**). The most significant difference in T_Naive_ was observed among the groups, with lower levels in Re-TB compared to Pr-TB (*P*<0.05) (**Figures 3B, F**), which prompted Re-TB patients may exhibit a reduced capacity to clear Mtb *in vivo*, resulting in more severe lung injury (**Supplementary Figure S1C**), and a higher risk for poor treatment outcomes (**Supplementary Figures S2B, C**). In contrast, there were no significant differences in T_CM_ between TB (Re-TB and Pr-TB) and non-TB (HC and RC), but Re-TB patients exhibited higher levels of T_CM_ cells compared to Pr-TB (*P*<0.05) (**Figures 3C, G**). Analysis of T_EM_ and T_RAEM_ cells revealed decreases in both subsets among TB patients, with significant differences observed only in CD8+ T_RAEM_ (**Figures 3D, E, H, I**).

### 3.3 Persistent stimulation by Mycobacterium tuberculosis leads to terminal differentiation of antigen-specific T cells

**Figure 4:**
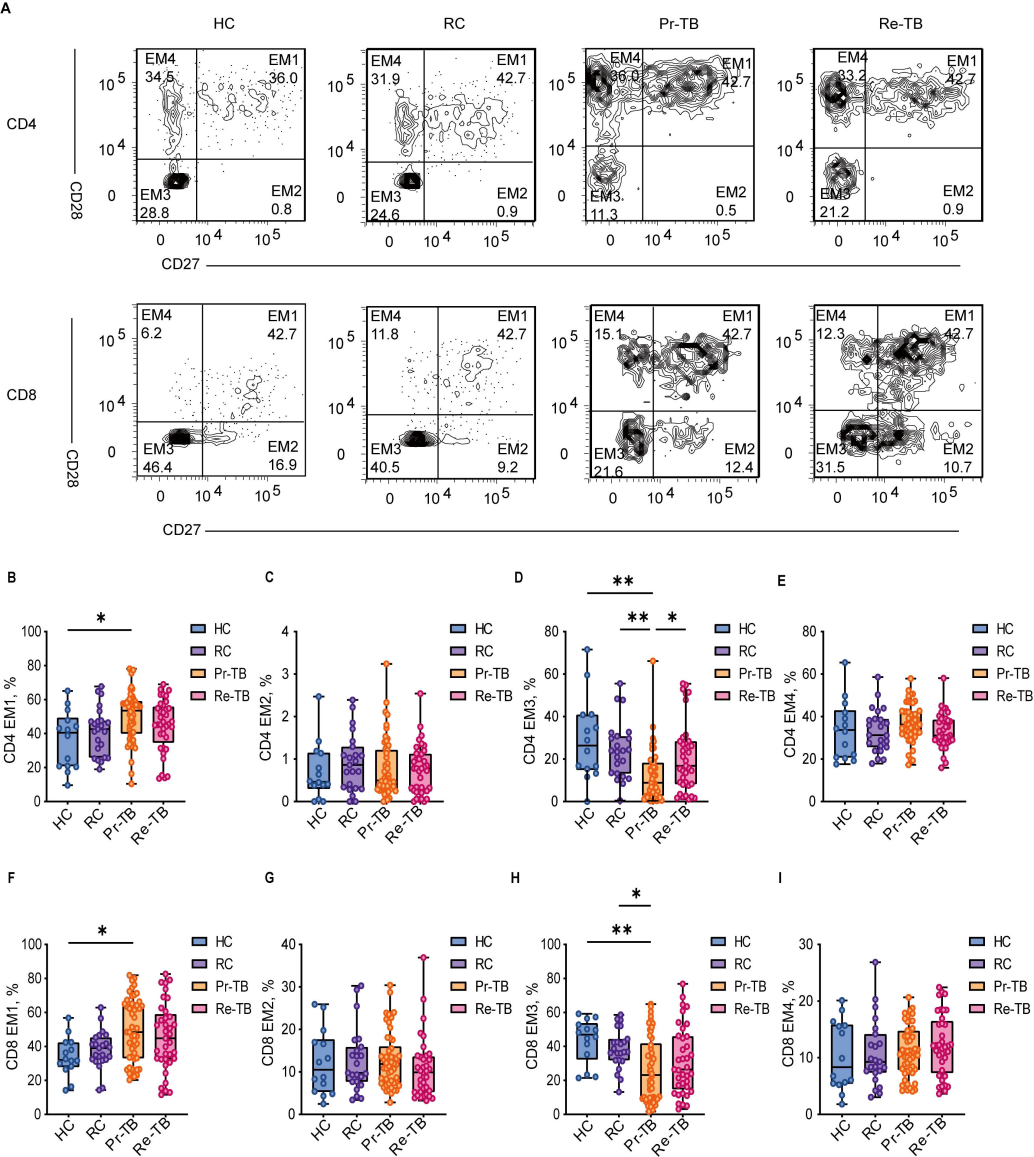
Frequency of EM CD4+ and CD8+ T lymphocyte subsets with different patterns of CD27 and CD28 expression in different groups. **(A)** Representative dot plots of EM1(CD27+CD28+), EM2(CD27+CD28-), EM3(CD27-CD28-), EM4(CD27-CD28+) lymphocyte subsets showing the mean frequencies of each study group. **(C–F)** Scatter plot showing the frequency comparison of CD4+ in different groups. **(G–J)** Scatter plot showing the frequency comparison of CD8+ in different groups. Significant differences are expressed as *<0.05, **<0.01, ***<0.001.

T cells exhibit the ability to differentiate into various functional states upon interaction with antigens. Prolonged antigenic stimulation leads to the functional impairment of both antigen-specific and non-specific T cells, which is characterized by differential expression of surface markers, including CD27, CD28, PD1, and CD57. In our analysis of T-cell activation in Re-TB, we employed a labeling strategy to classify four subsets of T_EM_ based on the expression of the CD27 and CD28 co-receptors (**Figure 4A**). The results indicated that in Pr-TB, a greater proportion of EM1 (CD27+CD28+) differentiated more rapidly compared to other subsets (**Figures 4B, F**) (*P*<0.05). In contrast, the terminally differentiated EM3 (CD27-CD28-) exhibited reduced differentiation (*P*<0.05) (**Figures 4D, H**). However, no significant differences were observed in T lymphocyte subsets in the Re-TB (**Figures 4C, E, G, I**).

**Figure 5:**
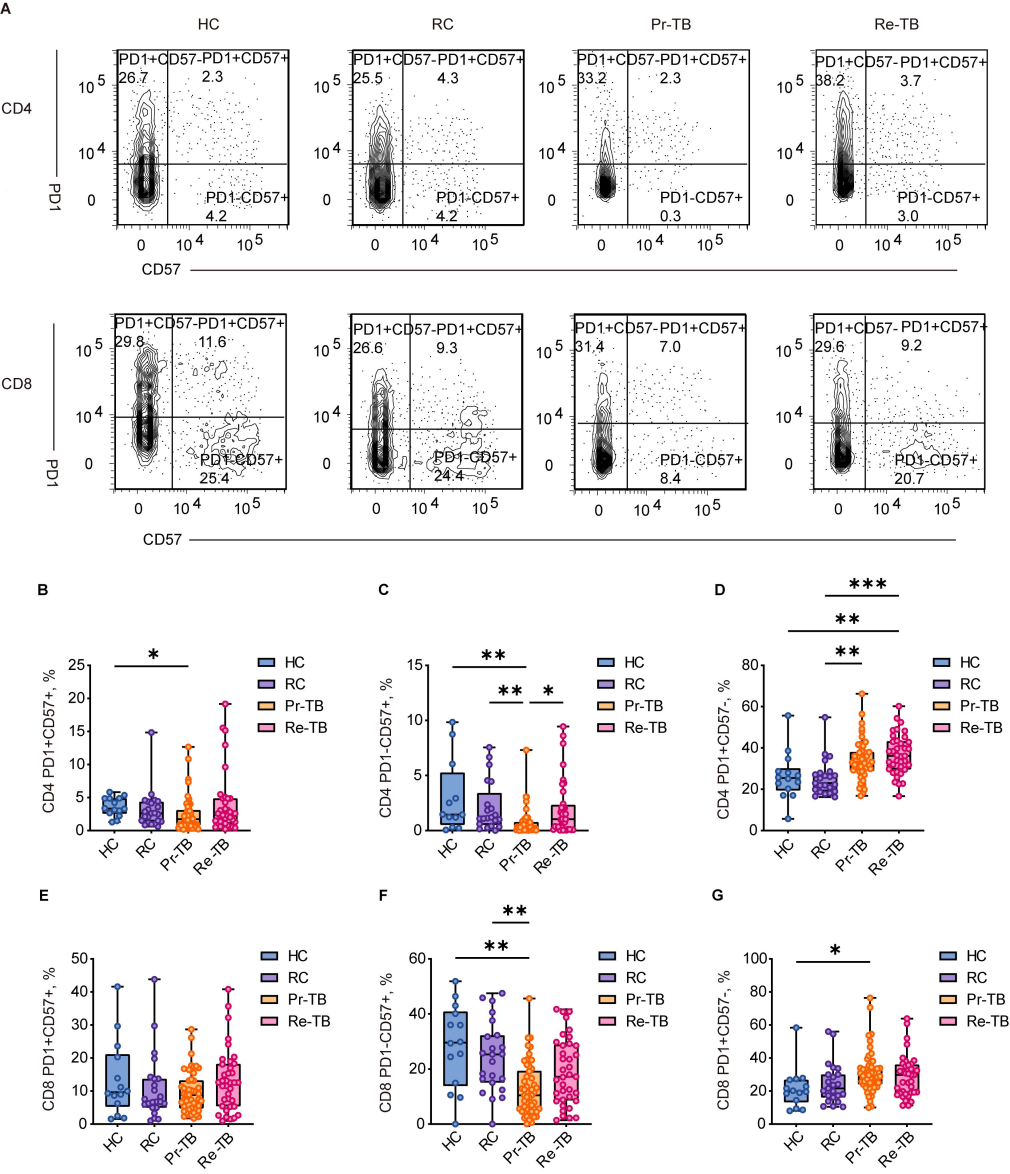
Frequency of RAEM CD4+ and CD8+ T lymphocyte subsets with different patterns of PD1 and CD57 expression in different groups. **(A)** Representative dot plots of PD1+CD57+,PD1-CD57+, PD1+CD57- showing the mean frequencies of each group. **(C–E)** Scatter plot showing the frequency comparison of CD4+ in different groups. **(F–H)** Scatter plot showing the frequency comparison of CD8+ in different groups. Significant differences are expressed as *<0.05, **<0.01, ***<0.001.

A population-based study revealed a significant prevalence of senescent cells in MDR, with notable increases in PD1 and CD57 expression in both CD4 and CD8 T cells (23). Further investigations into T-cell depletion in TB (**Figure 5A**), using PD1 and CD57 labeling, revealed that CD4 T cells in Re-TB exhibited significantly higher differentiation of PD1+CD57- cells (*P*<0.01) (**Figures 5B–G**), suggesting that CD4 T cells play a more prominent role in Re-TB.

### 3.4 T_Naive_ and T_CM_ as risk/protective factors for retreatment of patients

The distinct accumulation of T lymphocyte subsets observed across the four cohorts indicates different accumulation patterns in Re-TB compared to Pr-TB, suggesting varied T-cell immune activation profiles in these disease states. Patients with Re-TB exhibited a unique clinical profile relative to those with Pr-TB, as evidenced by significant differences in multiple disease severity-related variables (**Supplementary Table 4**). To identify potential risk or protective factors associated with retreatment, we performed a regression analysis on T lymphocyte subsets showing differential expression between the groups. We identified several key factors potentially contributing to retreatment (**Table 1**). Specifically, a reduction in T_Naive_ was associated with an increased risk of retreatment (adjusted OR=1.032, 95% CI: 1.001-1.064, *P*<0.05) (adjusted OR=1.039, 95% CI: 1.003-1.077, *P*<0.05) and an increase in T_CM_ cells was associated with a reduction risk (adjusted for age, drug resistance, adverse drug reactions, sputum smear, and lung injury) (adjusted OR=0.926, 95% CI: 0.860-0.996, *P*<0.05) (adjusted OR=0.951, 95% CI: 0.912-0.992, *P*<0.05).

**Table 1:** Analysis of the effect of T lymphocyte subsets on tuberculosis treatment (Pr-TB: n=50 and Re-TB: n=37).

	Crud OR(95%CI)	*P*-value	Model1		Model2		Model3	
			OR(95%CI)	*P*-value	OR(95%CI)	*P*-value	OR(95%CI)	*P*-value
CD8+ cells subsets								
Naive % Parent	1.026 (1.003,1.049)	0.026	1.028 (1.001,1.056)	0.043	1.031 (1.002,1.061)	0.037	1.032 (1.001,1.064)	0.044
RAEM % Parent	1.038 (1.002,1.075)	0.040	1.036 (0.994,1.083)	0.090	1.037 (0.992,1.085)	0.112	1.033 (0.985,1.084)	0.179
CM % Parent	0.925 (0.869,0.986)	0.016	0.927 (0.866,0.988)	0.021	0.923 (0.862,0.988)	0.021	0.926 (0.860,0.996)	0.040
CD4+ cells subsets								
Naive % Parent	1.034 (1.006,1.063)	0.016	1.042 (1.004,1.072)	0.027	1.040 (1.005,1.077)	0.025	1.039 (1.003,1.077)	0.034
CM % Parent	0.960 (0.931,0.990)	0.010	0.951 (0.914,0.988)	0.011	0.947 (0.909,0.987)	0.010	0.951 (0.912,0.992)	0.019
EM3% Parent	1.041 (1.008,1.075)	0.015	1.033 (0.994,1.073)	0.095	1.031 (0.990,1.073)	0.142	1.035 (0.989,1.075)	0.152
PD1-CD57+ % Parent	1.297 (1.021,1.647)	0.033	1.229 (0.944,1.600)	0.126	1.208 (0.993,1.565)	0.152	1.206 (0.934,1.557)	0.151

Crud OR is not adjusting.

Model1 is adjusting age, resistance and drug adverse vent.

Model2 is adjusting age, resistance, drug adverse vent and sputum smear.

Model3 is adjusting age, resistance, drug adverse vent, sputum smear, and lung injury.

### 3.5 An increase of T_CM_ improves the efficacy in Retreatment tuberculosis

**Figure 6:**
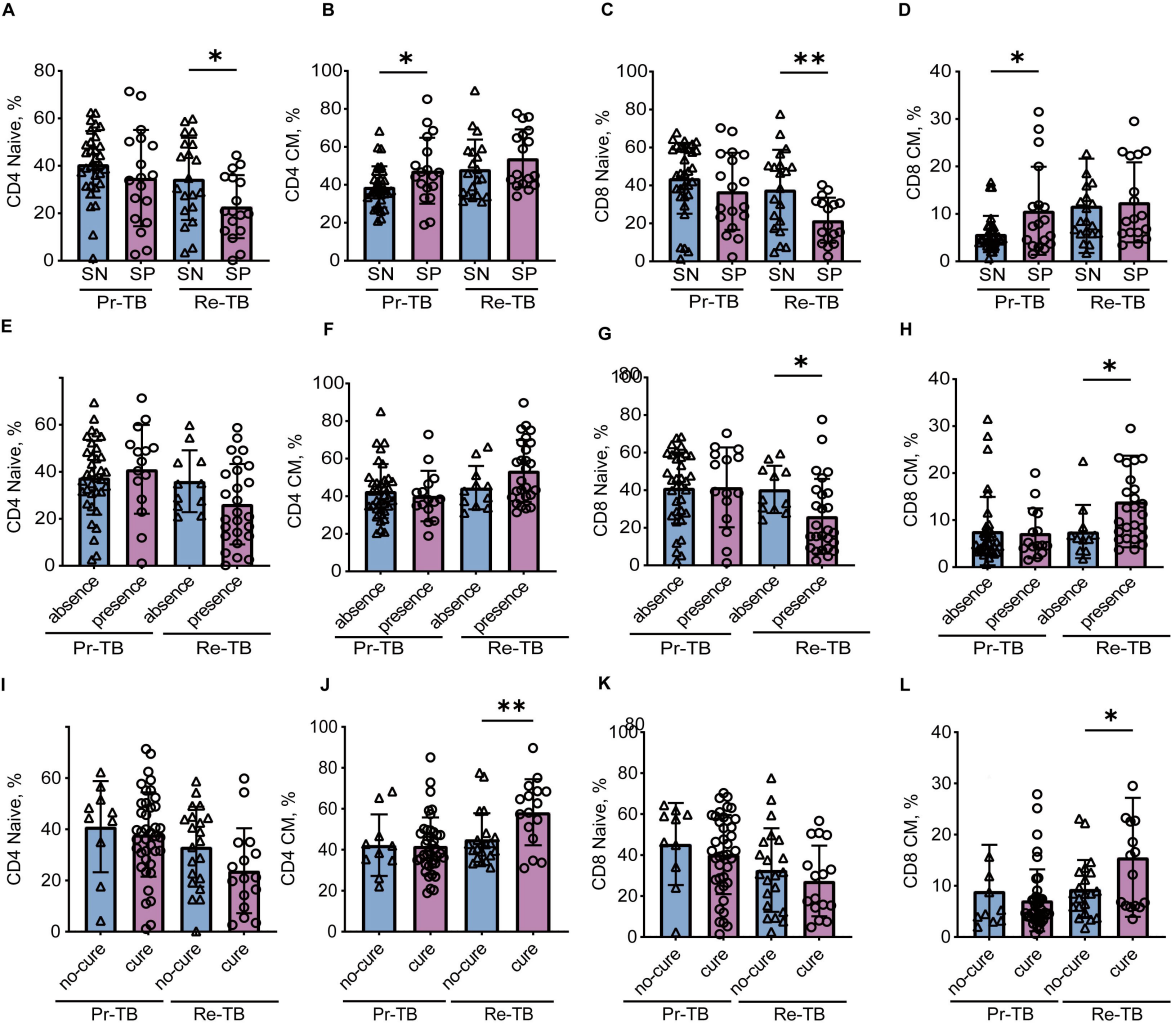
Comparison of T_Naive_ and T_CM_ between primary and retreatment tuberculosis in different situations. (**A-D)** Scatter plot showing the frequency comparison of T_Naive_ and T_CM_ between Pr-TB and Re-TB on the sputum smear. **(E-H)** Scatter plot showing the frequency comparison of T_Naive_ and T_CM_ between Pr-TB and Re-TB on the lung injury. (**I-L)** Scatter plot showing the frequency comparison of T_Naive_ and T_CM_ between Pr-TB and Re-TB on the cure. Two groups of comparative data follow a normal distribution using t-test, otherwise using Wilcoxon signed-rank test. Significant differences are expressed as *<0.05, **<0.01, ***<0.001. Pr-TB, Primary treatment tuberculosis (n=50); Re-TB, Retreatment tuberculosis (n=37).

A population-based study indicated that lung injury was associated with CD8 T cells, and that T lymphocyte subsets play a significant role in the transformation of sputum smear culture after treating two months of treatment. Studies have shown that T_CM_ cells are center to immunological memory, and may paly a role in Re-TB. We identified how T_CM_ cells affect Re-TB patients by controlling for sputum smear (SN and SP), lung injury (absense and presence), and treatment outcomes (cure and no-cure) (**Figure 6**) (**Table 2**). Our results showed that in Re-TB patients, T_Naive_ cells were significantly lower in those with a low bacterial load (*P*<0.05) (**Figures 6A, C**), that patients with lung injury exhibited a lower frequency of CD8+ T_Naive_ cells and a higher frequency of CD8+ T_CM_ cells (*P*<0.05) (**Figures 6G, H**).

**Table 2:** The impact of T_Naive_ and T_CM_ on the cure of tuberculosis in retreatment.

	Crud OR (95%CI)	*P*-value	Model1		Model2		Model3	
			OR (95%CI)	*P*-value	OR (95%CI)	*P*-value	OR (95%CI)	*P*-value
CD3+CD4+ cells subsets								
Naive % Parent	1.033 (0.990,1.077)	0.133	0.964 (0.907,1.026)	0.151	0.954 (0.902,1.008)	0.095	0.960 (0.898,1.025)	0.222
CM % Parent	0.939 (0.892,0.989)	0.016	0.926 (0.866,0.990)	0.024	0.920 (0.858,0.988)	0.022	0.890 (0.812,0.976)	0.013
CD3+CD8+ cells subsets								
Naive % Parent	1.016 (0.982,1.052)	0.354	1.008 (0.950,1.070)	0.797	0.967 (0.913,1.024)	0.244	0.973 (0.903,1.049)	0.479
CM % Parent	0.892 (0.806,0.989)	0.030	0.874 (0.771,0.990)	0.034	0.871 (0.767,0.990)	0.035	0.803 (0.677,0.953)	0.012

Crud OR is not adjusting.

Model1 is adjusting age, resistance and drug adverse vent.

Model2 is adjusting age, resistance, drug adverse vent and sputum smear.

Model3 is adjusting age, resistance, drug adverse vent, sputum smear, and lung injury.

Our study identified a relationship between T_Naive_ and T_CM_ and the outcome of Re-TB, there was no significant difference in T_Naive_ between patients who were cured and those who were not (**Figures 6I, K**); however, T_CM_ cells were significantly higher in the cured group (*P*<0.05) (**Figures 6J, L**). Furthermore, the adjusted model indicated that increased T_CM_ cells were associated with a greater likelihood of a cure in Re-TB (**Table 2**) (adjusted OR = 0.803, 95% CI: 0.677-0.953, *P*<0.05) (adjusted OR = 0.890, 95% CI: 0.812-0.976, *P*<0.05). Notably, the role of T_CM_ cells in Re-TB was not observed in Pr-TB, suggesting that increased T_CM_ in Re-TB may be associated with sustained long-term immune protection after the first infection, thereby controlling the progression of Mtb.

We have demonstrated in previous studies that T_CM_ can improve the efficacy in patients with Re-TB who experience repeated infections. To further ascertain the impact of the increased T_CM_ on the efficacy of Re-TB treatment, we re-assessed the treatment effects based on sputum smear results (SN and SP) and lung injury (absence and presence) (**Supplementary Figure 3**) (**Table 3**). The findings indicated that higher T_CM_ may enhance efficacy in patients with lower bacterial loads (*P*<0.05) (**Supplementary Figures 3A, B**) and in those with lung injury (*P*<0.05) (**Supplementary Figures 3C, D**). Furthermore, the adjusted regression model showed that higher T_CM_ treatment in patients with lung injury was associated with better efficacy (adjusted OR=0.780, 95%CI: 0.635-0.957, *P*<0.05) (adjusted OR=0.805, 95%CI: 0.660-0.983, *P*<0.05) (**Table 3**).

**Table 3:** The impact of T_CM_ on the cure of tuberculosis in retreatment with SN and lung injury.

	crud OR (95%CI)	*P*-value	Model1		Model2		Model3	
			OR (95%CI)	*P*-value	OR (95%CI)	*P*-value	OR (95%CI)	*P*-value
SN								
CD4+ CM % Parent	0.927 (0.853,1.007)	0.073	0.908 (0.789,1.046)	0.181	–	–	0.886 (0.761,1.031)	0.116
CD8+ CM % Parent	0.793 (0.634,0.990)	0.041	0.724 (0.535,0.980)	0.036	–	–	0.269 (0.069,1.059)	0.060
presence lung injury								
CD4+ CM % Parent	0.894 (0.822,0.971)	0.008	0.818 (0.676,0.989)	0.038	0.805 (0.660,0.983)	0.033	–	–
CD8+ CM % Parent	0.817 (0.694,0.962)	0.015	0.787 (0.646,0.958)	0.017	0.780 (0.635,0.957)	0.017	–	–

Crud OR is not adjusting.

Model1 is adjusting age, resistance and drug adverse vent.

Model2 is adjusting age, resistance, drug adverse vent and sputum smear.

Model3 is adjusting age, resistance, drug adverse vent, sputum smear, and lung injury.

## 4 Discussion

DR-TB and Re-TB are the challenge of TB prevention and treatment (1), and their coexistence is the main reason for lower cure rate of patients, which in our study cure rate for Re-TB (72.97% for DR-TB) was only 43.24%. Results found changes of some T lymphocyte subsets in Re-TB, especially an increase in T_CM_ cells, which have long-term immunologic memory against pathogens (10). By analyzing the different clinical characteristics of T_CM_ in Re-TB, results demonstrated that T_CM_ has a protective effect on treatment, and cured patients have higher T_CM_ levels, which we demonstrated T_CM_ cells could serve the long-term immune protective effect for Re-TB by establishing regression models for the first time. In brief, T_CM_ cells are long-term memory cells that could play a immune protection role in defending against Mtb reinfection. Enhancing T_CM_ cells may be a potential target for immunotherapy in Re-TB, offering significant reference for explore effective immunotherapy and developing protective vaccines.

The interaction between T cells and Mtb is a complex and prolonged process (24). Our study demonstrated that patients with Pr-TB and Re-TB exhibited higher levels of CD4 and CD8 T cells compared to non-TB individuals, consistent with previous research (25, 26), which implied the T-cell immunity in response to Mtb infection is maintained regardless of whether the infection long-term or short-term. Studies have showed that CD4 T cells were higher in active TB compared with LTBI (27, 28), which can serve as a diagnostic indicator for differentiating between them (29). The increased CD4 T cells may indicate improved immune function in TB patients (8), although it remains unclear whether these increased CD4 T cells in the cohort could secrete more cytokines, verifying in the next experiment. Study has shown that CD8 T cells correlate with the number of lung lobe lesions, with higher CD8 expression observed in patients with cavitary TB, suggesting that increased CD8 T cells are linked to lung injury (26, 30, 31). Our study also found increased HLA-DR expression on CD3 T cells in Re-TB patients, corroborating findings that HLA-DR serves as an activation marker in TB, indicated its expression enhances cytotoxic potential and cytokine secretion to promote control of Mtb infection and disease progression, it also indicated an increased risk of TB (8, 32). However, no significant differences in T lymphocyte subsets were found between Re-TB and Pr-TB, suggesting that patients with Re-TB do not exhibit a heightened capacity to eliminate Mtb; the results was also consistent in HC and RC.

Infection with Mtb triggers the recognition of structural molecular epitopes by T_Naive_, leading to their activation, proliferation, and differentiation into various subsets (33). During the initial phase of the adaptive immune response, exposure to antigens leads to the expansion and differentiation of antigen-specific T_Naive_ (33). The immunoprotective role of memory T cells is particularly crucial in TB patients with long-term repeated infection of Mtb. Studies have shown that patients with active TB exhibit higher levels of T_Naive_ and lower levels of T_CM_ compared to LTBI and healthy individuals (34, 34), the finding that was also true in our study. Notably, we observed that T_CM_ cells increased in Re-TB patients, which suggested that an increase in T_CM_ is an effective defense mechanism against Mtb and that T_CM_ can rapidly initiate a robust immune response upon re-exposure to the same antigen (15, 17). Research has indicated that co-regulatory molecules are upregulated during T-cell activation, and their co-expression is associated with T-cell depletion (27). Our study revealed a significant degree of CD4 T-cell depletion in TB, consistent with prior studies (35, 36), which the depletion during chronic infection is characterized by a progressive loss of proliferation, cytokine production, and cytotoxic T lymphocyte (CTL) activity (37).

Studies have found that increased T_CM_ can contribute to clear Mtb in mice (21, 37). Increased T_CM_ cells are widely recognized as a marker of reduced bacterial load and enhanced long-term antigen-specific memory T-cell responses (38). We analyzed the changes in T_CM_ by analyzing the activation of T cells in patients themselves for the first time. In our cohort, the median duration of treatment for Pr-TB was 12 months, while Re-TB patients were treated for a median of 18 months, indicating that Re-TB was characterized by a longer treatment duration, a more complex regimen, and poorer outcomes compared to Pr-TB. Differences in the expression profiles of T lymphocyte subsets between Re-TB and Pr-TB prompted the development of a regression model to identify potential risk factors associated with retreatment. Our analysis indicated that reduced T_Naive_ cells were linked to an elevated risk of retreatment, while increased T_CM_ cells appeared to mitigate the risk, confirming the long-term immune protective effect of T_CM_ in repeated infections. When controlling for various clinical characteristics in Re-TB patients, we found that T_Naive_ was significantly reduced in individuals with high sputum bacterial loads, corroborating previous studies reporting a negative correlation between bacterial loads and T lymphocyte subsets (39). Reinfection was associated with heightened immune dysregulation in Re-TB patients, many of whom presented with lung injuries, including cavity formation (40). Additionally, a decrease in CD8+ T_Naive_ was recorded in Re-TB patients with lung injury, while CD8+ T_CM_ increased, suggesting that these changes in the CD8 T cells were linked to the observed lung injury, consistent with findings from population-based studies (30, 41).

Interestingly, our results found that T_CM_ cells were significantly higher in cured patients, underscoring the pivotal role of T_CM_ in improving efficacy and cure rates in Re-TB (2, 4), confirmed by adjusting the statistical models, suggesting that augmenting T_CM_ could represent an effective treatment strategy for Re-TB management. In pharmacological studies of mouse models, the use of clofazimine to stimulate BCG resulted in an increase in T_CM_ (38), and combination therapy of clofazimine and rapamycin demonstrated better bactericidal effects in mice infected with drug-resistant bacteria, inducing a multifunctional T_CM_ response (21), conferring long-term protective immunity against TB (10, 35, 36). We observed that T_CM_ functioned less effectively in SN on Re-TB, and an increase in T_CM_ cells was observed in patients who were cured, but T_CM_ cells were similar between cured and no-cured Pr-TB; Our study suggests that in Re-TB, which was associated with a longer duration of infection (with a median relapse time of 3.5 years), the long-term immune protection provided by T_CM_ cells was more significant. Furthermore, increased T_CM_ cells were more effective in improving efficacy for patients with lung injury,likely due to the prolonged immune challenge posed by Mtb, which leads to sustained injury and, consequently, enhanced long-term protection by T_CM_ (18) (19). Adoptive immune therapy using T_CM_ has been shown to have therapeutic effects in treating infections and cancer, achieved through the *in vitro* expansion of T cells from the T_CM_ subset of donors (42). The role of immunotherapy in treating TB is increasingly recognized as vital, particularly for DR-TB and Re-TB, that is, targeting T_CM_ may provide a promising strategy for immunotherapy and development of protective vaccines in Re-TB, which suggest clinical immunological research should focus on enhancing T_CM_ in future to reduce treatment duration, mitigate tissue injury, and improve therapeutic efficacy.

Based on previous research findings (21, 38), we fistly further discovered in a population-study that T_CM_ is a protective factor for Re-TB patients and can predict the recovery status of patients for the first time, but there were several limitations in this study. The potential impact of TB drugs on the results related to T_CM_ cannot be entirely excluded. The lack of ongoing monitoring of changes in T_CM_ cells in patients throughout the study limits the understanding of their dynamics. The study relied on predictive models to assess the relationship between T_CM_ and treatment outcomes, rather than direct observations or interventions. Additionally, the findings are based on a specific population, which may not be generalizable to all Re-TB patients. There was insufficient exploration of the activation mechanisms, and data were only collected once during the entire study. Moreover, differences in treatment protocols among participants could introduce variability, potentially affecting the study’s findings. Despite these limitations, our study demonstrates the potential of T_CM_ as a target for immunotherapy in Re-TB and highlights the need for future research to explore this promising approach.

In conclusion, T_CM_ cells can serve as a protective factor in Re-TB patients, predicting recovery statuses and playing a key role in long-term immune protection against TB reinfection. Therefore, T_CM_ could represent a promising immunotherapeutic target, particularly in the development of protective vaccines, and serve as a focus for new drug development for Re-TB, which highlights the critical role of T_CM_ in managing disease progression and treating Re-TB, while also paving the way for future research in Re-TB immunotherapy, thereby contributing valuable insights to both the treatment of Re-TB and the development of novel therapeutic strategies.

## Data Availability

The original contributions presented in the study are included in the article/**Supplementary Material**. Further inquiries can be directed to the corresponding author.
