# Supplementary material for: Increased long-term central memory T cells in patients with retreatment pulmonary tuberculosis
Source: Front Immunol. 2025 Mar 18;16:1545537. doi: 10.3389/fimmu.2025.1545537 (PMC11959053; doi:10.3389/fimmu.2025.1545537)
Supplement: Supplementary file 1 [file DataSheet1.docx]

Supplementary Material

# Supplementary Figures and Tables

## Supplementary Figures


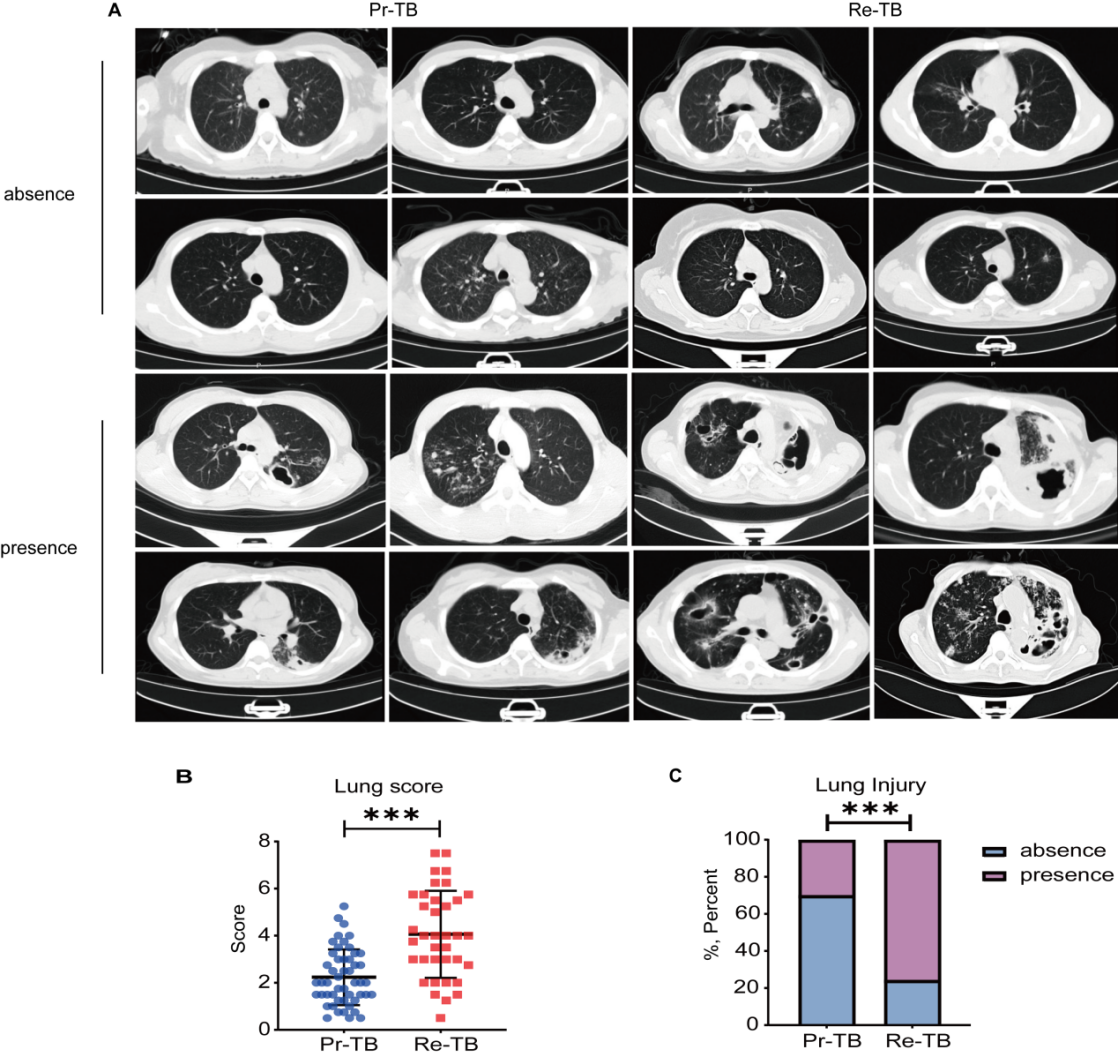


**Supplementary Figure 1.** CT imaging and scoring of Pr-TB and Re-TB. A: The lung CT images of Pr-TB and Re-TB were displayed separately, with absence indicating the absence of lung injury and presence indicating the presence of lung injury. B: Comparison of CT scoring results between two groups was presented. C: Compare the situation of lung injury between two groups. Two groups of comparative data follow a normal distribution using t-test and χ2 test. Significant differences are expressed as *<0.05, **<0.01, ***<0.001. Pr-TB =Primary treatment tuberculosis (n=50); Re-TB = Retreatment tuberculosis (n=37).

**
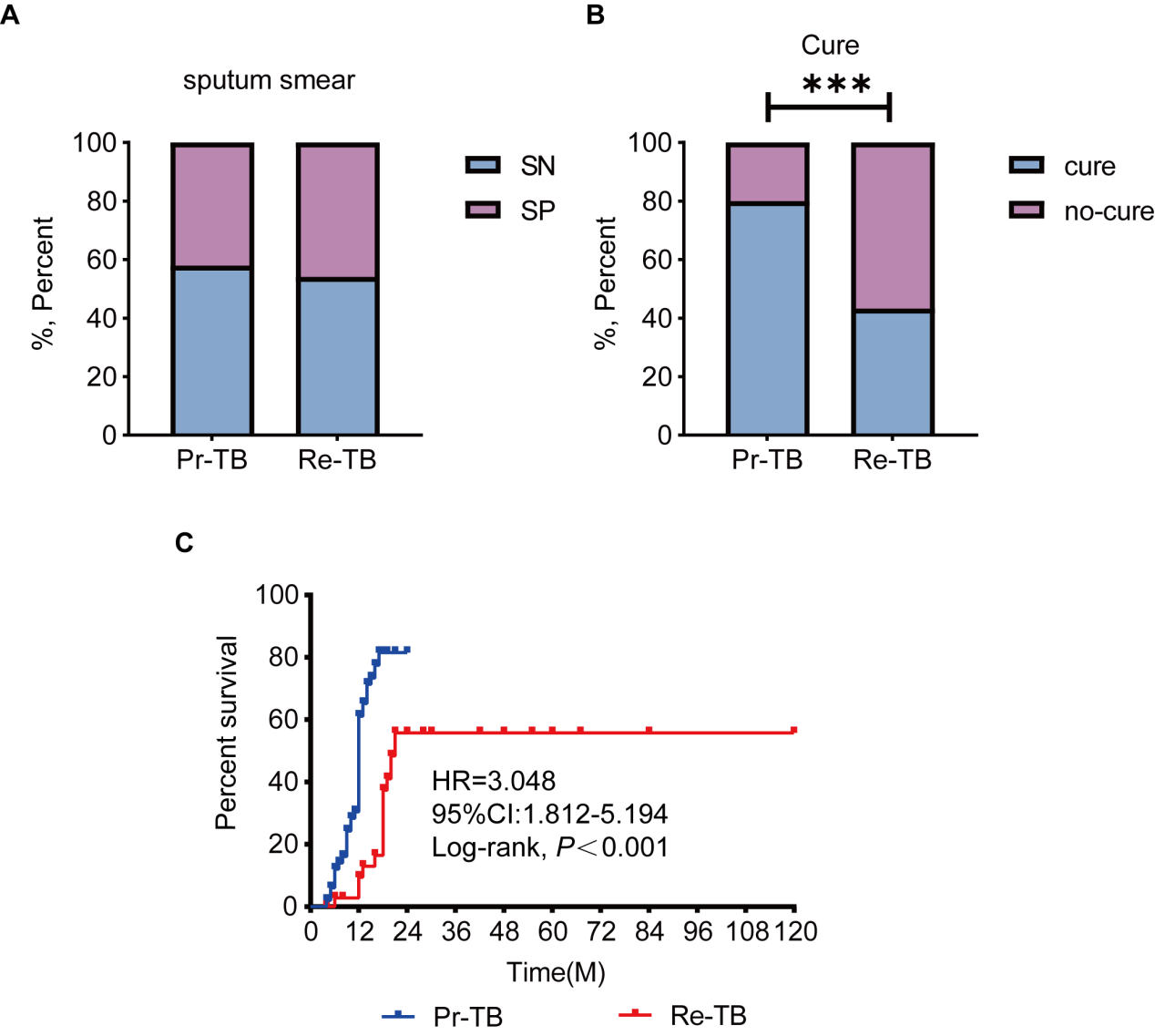
**

**Supplementary Figure 2.** Comparison of treatment status between Pr-TB and Re-TB. A: showing the sputum smear. B: showing the cure. C: showing the survival curves. Data composition ratio is represented by rate, and statistical analysis is performed by χ2 test Significant differences are expressed as *<0.05, **<0.01, ***<0.001. Pr-TB = Primary treatment tuberculosis (n=50); Re-TB = Retreatment tuberculosis (n=37).

**
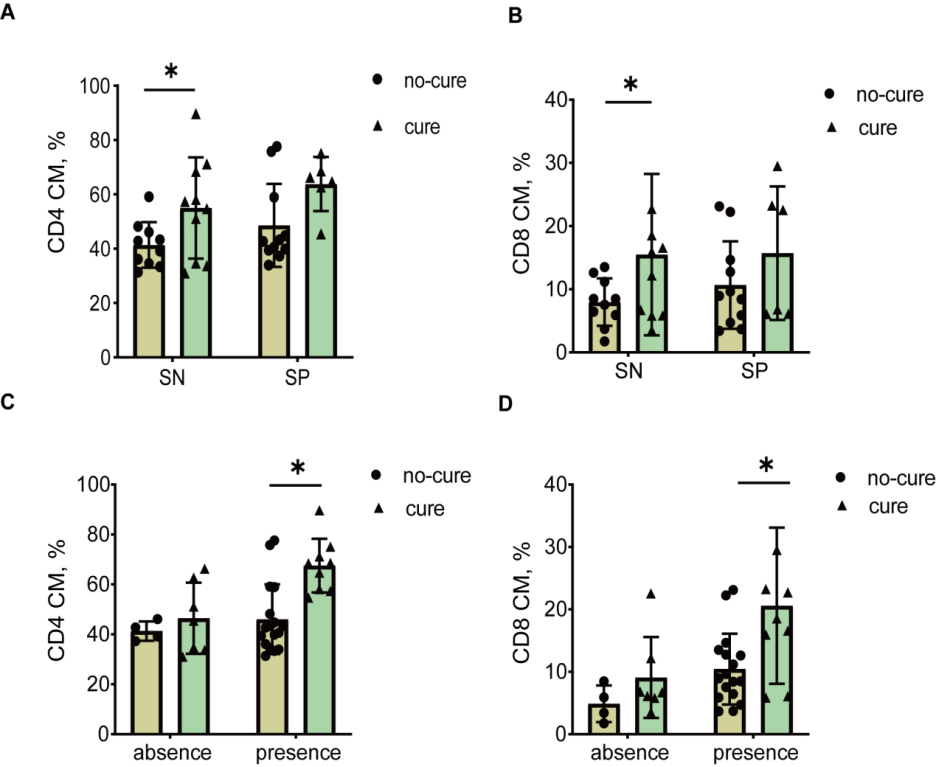
**

**Supplementary Figure 3.** Comparison of TCM in Re-TB on the sputum smear and lung injury. A-B: Scatter plot showing the frequency comparison of TCM in Re-TB on the sputum smear. C-D: Scatter plot showing the frequency comparison of TCM in Re-TB on the lung injury. Two groups of comparative data follow a normal distribution using t-test, otherwise using Wilcoxon signed-rank test. Significant differences are expressed as *<0.05, **<0.01, ***<0.001.

## Supplementary Tables

| **Table S1.**  Baseline characteristics of all research subjects（N=124） | | | | | | |
| --- | --- | --- | --- | --- | --- | --- |
| characteristics |  | HC (n=14) | RC (n=23) | Pr-TB  （n=50） | Re-TB  （n=37） |  |
| Sex |  |  |  |  |  |  |
| male |  | 11（78.57） | 10（43.48） | 28（56.00） | 23（62.16） |  |
| female |  | 3（21.43） | 13（56.52） | 22（44.00） | 14（37.84） |  |
| Age |  |  |  |  |  |  |
| Mean |  | 30.1（21-44） | 58.4（42-74） | 35.7（18-70） | 46.4（19-71） |  |
| BMI |  |  |  |  |  |  |
| ≤24 |  | 9（62.29） | 15（65.22） | 42（84.00） | 24（64.86） |  |
| ＞24 |  | 5（35.71） | 8（34.78） | 8（16.00） | 13（35.14） |  |
| Xpert |  |  |  |  |  |  |
| positve |  | 0 | 0 | 50（100） | 37（100） |  |
| negative |  | 14（100） | 23（100） | 0 | 0 |  |

| **Table S2.**  Pulmonary scoring criteriaent. | | |
| --- | --- | --- |
| CT manifestations |  | score |
| Injury site |  |  |
| Both lungs |  | 0.5 |
| Left /right lung |  | 0.25 |
| Damage area |  |  |
| 0% |  | 0 |
| 25% |  | 0.25 |
| 50% |  | 0.5 |
| 75% |  | 0.75 |
| 100% |  | 1 |
| Pleural thickening |  | 0.5 |
| cavity |  | 1 |
| pleural effusion |  | 1 |
| Patchy shadow |  | 0.5 |
| tubercle |  | 0.5 |
| Lymphnode enlargement |  | 0.25 |
| Irregular shape |  | 0.25 |
| Calcified lesion |  | 0.5 |
| emphysema |  | 1 |
| Pulmonary bulla |  | 1 |
| Unclear boundaries |  | 0.25 |
| Lung destruction |  | 2 |
| White lung |  | 2 |
| Atelectasis |  | 1 |

| **Table S3.**  Flow cytometry analysis markers for T lymphocyte subsets. | | | | |
| --- | --- | --- | --- | --- |
| T cells subsets | surface marker | | FSC | SSC |
|  | Specificity | Purpose |  |  |
| CD3+ | CD3 | T cells | CD3 R763-APCA750-A | SSC-A |
| CD4+ | CD4 | Helper T cells |  | CD4 R660-APC-A |
| CD8+ | CD8 | Cytotoxic T cells |  | CD8 R712-APCA700-A |
| HLA-DR | HLA-DR | T cell differentiation |  | HLA-DRV763-A |
| Naive | CD45、CCR7 | T cell differentiation | CCR7 Y585-PE-A | CD45RAB525-FITC-A |
| RAEM |  |  |  |  |
| EM |  |  |  |  |
| CM |  |  |  |  |
| CD28+CD27+ | CD27、CD28 | T, B, and NK cell differentiation | CD27 Y763-PC7-A | CD28 B610-ECD-A |
| CD28+CD27- |  |  |  |  |
| CD28-CD27- |  |  |  |  |
| CD28-CD27+ |  |  |  |  |
| PD1+CD57+ | CD57、PD1 | NK and T cell differentiation | CD57 V450-PB-A | PD-1 Y710-PC5.5-A |
| PD1+CD57- |  |  |  |  |
| PD1-CD57+ |  |  |  |  |

| **Table S4.**  Baseline demographic characteristics and clinical characteristics for patients( Pr-TB: n=50；Re-TB: n=37). | | | | | | | | |
| --- | --- | --- | --- | --- | --- | --- | --- | --- |
|  | Total (n=87) | | Pr-TB （n=50） | | Re-TB  （n=37） | |  | P-value |
| Sex |  |  |  |  |  |  |  | 1.000 |
| male | 51 | 58.62 | 28 | 56.00 | 23 | 62.16 |  |  |
| female | 36 | 41.38 | 22 | 44.00 | 14 | 37.84 |  |  |
| Age |  |  |  |  |  |  |  | 0.002 |
| ＜35 | 47 | 54.02 | 35 | 70.00 | 12 | 32.43 |  |  |
| 35-55 | 18 | 20.69 | 6 | 12.00 | 12 | 32.43 |  |  |
| ＞55 | 22 | 25.29 | 9 | 18.00 | 13 | 35.14 |  |  |
| BMI |  |  |  |  |  |  |  | 0.069 |
| ≤24 | 66 | 75.86 | 42 | 84.00 | 25 | 67.57 |  |  |
| ＞24 | 21 | 24.14 | 8 | 16.00 | 12 | 32.43 |  |  |
| Types of clinical symptoms |  |  |  |  |  |  |  | 0.029 |
| 0 | 28 | 32.18 | 21 | 42.00 | 7 | 18.92 |  |  |
| 1 | 39 | 44.84 | 23 | 46.00 | 16 | 43.24 |  |  |
| 2 | 10 | 11.49 | 3 | 6.00 | 7 | 18.92 |  |  |
| ≥3 | 10 | 11.49 | 3 | 6.00 | 7 | 18.92 |  |  |
| complication |  |  |  |  |  |  |  |  |
| hypertension | 7 | 8.05 | 2 | 4.00 | 5 | 13.51 |  | 0.112 |
| diabetes | 13 | 14.94 | 4 | 8.00 | 9 | 24.32 |  | 0.065 |
| anemia | 29 | 33.33 | 14 | 28.00 | 15 | 40.54 |  | 0.165 |
| sputum smear |  |  |  |  |  |  |  | 0.490 |
| negative | 52 | 56.77 | 32 | 64.00 | 20 | 54.05 |  |  |
| positive | 35 | 40.23 | 18 | 36.00 | 17 | 45.95 |  |  |
| CT imaging |  |  |  |  |  |  |  |  |
| cavity | 29 | 33.33 | 9 | 18.00 | 20 | 54.05 |  | 0.001 |
| pleural effusion | 13 | 14.94 | 9 | 18.00 | 4 | 10.81 |  | 0.549 |
| Grade of lung injury |  |  |  |  |  |  |  | 0.003 |
| light（＜3） | 46 | 52.87 | 35 | 70.00 | 9 | 24.32 |  |  |
| middle（3-） | 25 | 28.74 | 11 | 22.00 | 13 | 35.14 |  |  |
| weight（≥5) | 20 | 22.99 | 4 | 8.00 | 15 | 40.54 |  |  |
| Area of lung damage |  |  |  |  |  |  |  | 0.028 |
| left/right | 34 | 39.08 | 24 | 48.00 | 10 | 27.03 |  |  |
| both | 53 | 60.92 | 26 | 52.00 | 27 | 72.97 |  |  |
| Occurrence of adverse drug reaction events | 33 | 37.93 | 10 | 20.00 | 23 | 62.16 |  | 0.001 |
| drug-resistant | 39 | 44.83 | 12 | 24.00 | 27 | 72.97 |  | ＜0.001 |
| cure | 56 | 64.37 | 40 | 80.00 | 16 | 43.24 |  | 0.001 |

| **Table S5.**  The impact of T_Naive_ and T_CM_ on the sputum smear of tuberculosis in retreatment. | | | | | | | | | | |
| --- | --- | --- | --- | --- | --- | --- | --- | --- | --- | --- |
|  | crud OR(95%CI) | *P*-value | Model1 | |  | Model2 | |  | Model3 | |
|  |  |  | OR(95%CI) | *P*-value |  | OR(95%CI) | *P*-value |  | OR(95%CI) | *P*-value |
|  |  |  |  |  |  |  |  |  |  |  |
| CD3+CD4+ cells subsets |  |  |  |  |  |  |  |  |  |  |
| Naive % Parent | 0.953（0.929,1.010） | 0.038 | 0.964（0.907,1.026） | 0.151 |  | - | - |  | 0.949（0.901,0.999） | 0.047 |
| CM % Parent | 1.024（0.982,1.069） | 0.261 | 1.045（0.948,1.152） | 0.373 |  | - | - |  | 1.060(0.965,1.163) | 0.223 |
| CD3+CD8+ cells subsets |  |  |  |  |  |  |  |  |  |  |
| Naive % Parent | 0.947（0.905,0.990） | 0.017 | 0.942（0.891,0.996） | 0.034 |  | - | - |  | 0.938（0.884,0.996） | 0.037 |
| CM % Parent | 1.013（0.945,1.086） | 0.711 | 0.913（0.764,1.061） | 0.316 |  | - | - |  | 0.934（0.790,1.105） | 0.426 |
|  |  |  |  |  |  |  |  |  |  |  |

Crud OR is not adjusting;

Model1 is adjusting age, resistance and drug adverse vent;

Model2 is adjusting age, resistance, drug adverse vent and sputum smear;

Model3 is adjusting age, resistance, drug adverse vent, sputum smear, and lung injury.

| **Table S6.**  The impact of TNaive and TCM on the lung injury of tuberculosis in retreatment. | | | | | | | | | | |
| --- | --- | --- | --- | --- | --- | --- | --- | --- | --- | --- |
|  | crud OR(95%CI) | *P*-value | Model1 | |  | Model2 | |  | Model3 | |
|  |  |  | OR(95%CI) | *P*-value |  | OR(95%CI) | *P*-value |  | OR(95%CI) | *P*-value |
|  |  |  |  |  |  |  |  |  |  |  |
| CD3+CD4+ cells subsets |  |  |  |  |  |  |  |  |  |  |
| Naive % Parent | 0.969（0.927,1.013） | 0.164 | 0.993（0.932,1.057） | 0.815 |  | 0.990（0.928,1.056） | 0.757 |  | - | - |
| CM % Parent | 1.033（0.983,1.087） | 0.199 | 0.984（0.890,1.087） | 0.749 |  | 0.981（0.885,1.088） | 0.718 |  | - | - |
| CD3+CD8+ cells subsets |  |  |  |  |  |  |  |  |  |  |
| Naive % Parent | 0.967（0.931,1.004） | 0.078 | 0.980（0.925,1.038） | 0.487 |  | 0.978（0.922,1.037） | 0.450 |  | - | - |
| CM % Parent | 1.094（0.971,1.232） | 0.139 | 1.094（0.897,1.335） | 0.375 |  | 1.099（0.896,1.349） | 0.364 |  | - | - |
|  |  |  |  |  |  |  |  |  |  |  |

Crud OR is not adjusting;

Model1 is adjusting age, resistance and drug adverse vent;

Model2 is adjusting age, resistance, drug adverse vent and sputum smear;

Model3 is adjusting age, resistance, drug adverse vent, sputum smear, and lung injury.
